# Economic Impact of European Liver and Intestine Transplantation Association (ELITA) Recommendations for Hepatitis B Prophylaxis After Liver Transplantation

**DOI:** 10.3389/ti.2023.10954

**Published:** 2023-01-30

**Authors:** Paolo Angelo Cortesi, Raffaella Viganò, Sara Conti, Ilaria Lenci, Riccardo Volpes, Silvia Martini, Mario Angelico, James Fung, Maria Buti, Audrey Coilly, Francois Durand, Constantino Fondevila, Pascal Lebray, Frederik Nevens, Wojciech G. Polak, Mario Rizzetto, Fabien Zoulim, Giovanni Perricone, Marina Berenguer, Lorenzo Giovanni Mantovani, Christophe Duvoux, Luca Saverio Belli

**Affiliations:** ^1^ Research Centre on Public Health (CESP), University of Milano-Bicocca, Monza, Italy; ^2^ Hepatology and Gastroenterology Unit, ASST GOM Niguarda, Milan, Italy; ^3^ Unit of Hepatology and Liver Transplant Unit, Tor Vergata University, Rome, Italy; ^4^ Hepatology Unit, Department for the Treatment and Study of Abdominal Diseases and Abdominal Transplantation, IRCCS-ISMETT (Istituto Mediterraneo per I Trapianti e Terapia ad Alta Specializzazione), Palermo, Italy; ^5^ Gastro-hepatology Unit, Azienda Ospedaliera Universitaria, Città della Salute e della Scienza di Torino, University of Torino, Torino, Italy; ^6^ Hepatology and Liver Transplant Unit, University of Tor Vergata, Rome, Italy; ^7^ Department of Medicine, School of Clinical Medicine, Queen Mary Hospital, State Key Laboratory of Liver Research, The University of Hong Kong, Hong Kong, Hong Kong SAR, China; ^8^ Liver Unit, Hospital Universitario Valld’Hebron, Barcelona, Spain; ^9^ Centro de Investigación Biomédica en Red de Enfermedades Hepáticas y Digestivas (CIBEREHD), Instituto de Salud Carlos III, Madrid, Spain; ^10^ AP-HP Hôpital Paul-Brousse, Centre Hépato-Biliaire, Villejuif, France; ^11^ Unité INSERM 1193, Université Paris-Saclay, Paris, France; ^12^ Hepatology and Liver Intensive care, Hospital Beaujon, Clichy, France; ^13^ Service d’Hépatologie et Transplantation Hépatique, APHP, Hôpital Beaujon, Université Paris Diderot, INSERM U1149, Clichy, France; ^14^ HPB Surgery and Transplantation, Hospital Universitario La Paz, Instituto de Investigación Hospital Universitario La Paz (IdiPAZ), CIBERehd, Madrid, Spain; ^15^ Médecine Sorbonne Université, Service d’Hépato-gastroentérologie, Hôpitaux Universitaires Pitié Salpêtrière—Charles Foix, Groupe Hospitalier Pitié-Salpêtrière, Paris, France; ^16^ Division of Hepatology and Liver Transplantation, European Reference Network on Hepatological Diseases (ERN Rare-Liver), University Hospitals KU, Leuven, Belgium; ^17^ Erasmus MC, Transplant Institute, University Medical Center Rotterdam, Department of Surgery, Division of HPB and Transplant Surgery, Rotterdam, Netherlands; ^18^ Department of Medical Sciences, School of Medicine, University of Turin, Turin, Italy; ^19^ INSERM U1052—Cancer Research Center of Lyon (CRCL), Lyon University, Hospices Civils de Lyon, Lyon, France; ^20^ Hepatology and Liver Transplantation Unit, Ciberehd; Faculty of Medicine, La Fe University Hospital, Valencia, Spain; ^21^ Service d'Hépatologie, Hôpitaux Universitaires Henri Mondor, Créteil, France

**Keywords:** prophylaxis, hepatitis B, immunoglobulin (IgG), liver transplant, economics

## Abstract

The European Liver and Intestine Transplant Association, ELITA, promoted a Consensus Conference involving 20 experts across the world which generated updated guidelines on HBV prophylaxis in liver transplant candidates and recipients. This study explores the economic impact associated with the implementation of the new ELITA guidelines. To this aim, a condition-specific cohort simulation model has been developed to compare new and historical prophylaxis, including only pharmaceutical cost and using the European perspective. The target population simulated in the model included both prevalent and incident cases, and consisted of 6,133 patients after the first year, that increased to 7,442 and 8,743 patents after 5 and 10 years from its implementation. The ELITA protocols allowed a cost saving of around € 235.65 million after 5 years and € 540.73 million after 10 years; which was mainly due to early HIBG withdrawal either after the first 4 weeks or after the first year post Liver Transplantation (LT) depending on the virological risk at transplantation. Results were confirmed by sensitivity analyses. The money saved by the implementation of the ELITA guidelines would allow healthcare decision makers and budget holders to understand where costs could be reduced and resources re-allocated to different needs.

## Graphical Abstract

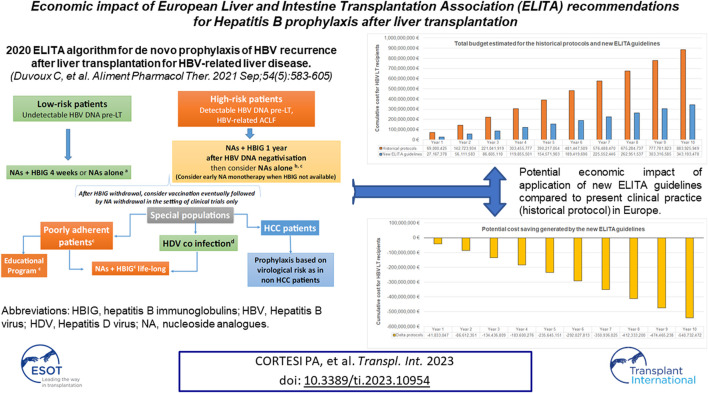

## Introduction

Prophylaxis for HBV recurrence is of critical importance post liver transplantation (LT). Despite the efficacy of new prophylactic regimens based on short term Hepatitis B Immunoglobulins (HBIG) use [1,2], most European LT centers persist with a conservative approach, combining hepatitis B immunoglobulin (HBIG) long term with nucleos(t)ides analogues (NA). The recently published ELITA guidelines provide updated new evidence that prophylactic strategies based on a personalized use of HBIG is possible; its duration dependent on the virological risk profile at the time of LT [3].

Based on this new approach there is the potential for substantial cost-savings to the healthcare budget, which could be reinvested in other areas to improve patient management and outcomes. To better understand the possible economic impact of these new strategies we performed a budget impact analysis (BIA) using the European perspective. This analysis aims at understanding the possible cost savings associated with the implementation of the new ELITA guidelines compared to current clinical practice.

## Materials and Methods

BIAs are increasingly required by budget holders and Healthcare Authorities to understand the economic impact of adopting a new healthcare intervention/treatment protocol in a specific population. BIA addresses the expected changes in the expenditure of a healthcare system after the adoption of a new intervention proving valuable information for budget or resource planning [4]. The computing framework for a BIA can be a simple cost calculator programmed on a spreadsheet or a condition-specific cohort or individual simulation model [4].

In this study, a condition-specific cohort simulation model was developed to compare new and historical treatment protocols, including only pharmaceutical cost and using the European perspective. The model estimates the cost of two different scenarios: Historical scenario, based on long term use of HBIG, and ELITA scenario, based on individualized use of HBIG according to the ELITA Clinical Practice Guidelines [3]. The model assumed that all patients in the historical scenario received long term HBIG, while patients in the ELITA scenario were treated according to the individualized virological risk at LT. The analysis was conducted using a 10-year time horizon.

The treatment protocols included in the two scenarios were.

1. *Historical protocol*
HBIG 5.000 IU/day intravenous IV) for 7 days +5.000 IU IV every 2 months life-long + NA lifelong.
2. *ELITA protocol (ELITA guidelines)*
*Low risk populations (HBV DNA negative at LT)*.
HBIG 5.000 IU/d IV for 7 days + NA lifelong.
*High risk population (HBV DNA positive at LT).*
HBIG 5.000 IU/day IV for 7 days + 5.000 IU IV every 2 months for 1year + NA life-long.
*Special population* (poorly adherent and HBV/HDV):
  HBIG 5.000 IU/day IV for 7 days +5.000 IU IV every 2 months life-long + NA lifelong.

Notably, patients transplanted, with hepatocellular carcinoma (HCC), were not considered as a special population but received a prophylactic regimen based on their virological risk, as patients with decompensated cirrhosis. Further, the dose of HIBG in the 7 days post-transplant theraphy was set at 5,000 IU/kg instead of 10,000 IU/Kg as reported in ELITA guidelines. This change in treatment protocol was based on the actual treatment performed in the majority of European LT centers. However, this change has no effect on the overall budget impact because the first 7 days treatment with HBIG post-transplant is the same in both historical and ELITA protocol (HBIG 5.000 IU/day intravenous IV) for 7 days).

The population simulated for this analysis consisted of all HBV transplanted patients performed in Europe over a 10-year time period. Patients were stratified into two groups: 1. Incident LT patients, all HBV patients forecast to receive a LT in the next 10 years, and 2. Prevalent LT patients, all alive patients transplanted in the last 15 years. The clinical and epidemiological data used in the model is reported in Table 1 and was based on the European Liver Transplant Registry (ELTR) data [5]. The number of incident cases, that is new HBV LT patients, were estimated assuming 506 cases per year, and a survival probability of 86.22% after 1 year, 77.77% after 5 years, and 71.43% after 10 years [5]. The number of prevalent cases, that is historical LT patients, were assumed to be 5,627, based on 7,593 HBV LT reported by Adams et al. over the last 15 years, with the associated survival probability [5].

**TABLE 1:** Model data input.

Parameters			Value (range)	References
*Epidemiological data*				
New yearly (incident) cases of HBV LT patients, N			506 (405–607)	Adam 2018
Number of prevalent HBV LT patients, N			5,627 (4,501–6,752)	Adam 2018
*Clinical data*				
*New LT patient*				
HBV liver transplant mortality probability at 1 year, %			13.78%	Adam 2018
HBV liver transplant mortality probability at 5 years, %			12.23%	Adam 2018
HBV liver transplant mortality probability at 10 years, %			18.57%	Adam 2018
*Prevalent LT patient*				
HBV liver transplant mortality probability at 5 years, %			6.34%	Adam 2018
HBV liver transplant mortality probability at 10 years, %			14.51%	Adam 2018
*Distribution of patients category included in ELITA protocol*				
Low risk			82.0%	Adam 2018
High risk			10.0%	Fraser 2013
Special patients			8.0%	Ladin 2018
*Treatment cost*				
Entecavir, € per mg			11.9 € (7.3–16.2)	Duvoux 2021
Tenofovir, € per 245 mg			8.4 € (4.4–13.6)	
HBIG IV, € per 5000 IU			1,589.6 € (1,029–2,772.7)	
Historical protocol		1st treatment year	24,366 €	Estimated
		≥2nd treatment year	13,239 €	
New protocol	Low risk^a^	1st treatment year	14,828 €	
		≥2nd treatment year	3,701 €	
	High risk^b^	1st treatment year	24,366 €	
		≥2nd treatment year	3,701 €	
	Special population^c^	1st treatment year	24,366 €	
		≥2nd treatment year	13,239 €	

^a^ Low virological risk patients: patients with undetectable HBV DNA, pre-LT, irrespective of Lt indication (cirrhosis or fulminant hepatitis).

^b^ High virological risk patients: Patients with detectable HBV DNA, at LT, Patients with HBV, reactivation resulting in HBV-related acute on chronic liver failure.

^c^ Special populations: Patients with HDV, co-infection, at low virological risk but deserving full prophylaxis, HCC, patients, at higher virological risk in case of HCC, recurrence but not requiring, patients at risk of poor adherence to antiviral therapy post-LT.

The predictive model assumed a survival probability of prevalent LT patients equal to 93.56% after 5 years and 85.49% after 10 years [5]. Furthermore, to define the number of patients associated to each category of the ELITA treatment protocol, the model had 82% prevalence of low-risk patients, 10% of high risk and 8% of special patients [5,6,7].

The pharmaceutical costs for HBV drugs were the only costs included in the analysis. The unit price applied to each drug was the average of drugs price reported in Spain, Italy, France, Austria, Belgium and Poland [3]. NA cost applied in the analysis was the average price of ENT (11.9 € per mg) and TDF (8.4 € per 245 mg). The price applied to HIBG was the one associated to the IV formulation (Table 1).

Based on these drug costs, the model estimated the total annual cost per patient per single treatment. The treatment cost per patient for the first year and the subsequent years are reported in Table 1. The estimated annual costs were combined with the epidemiological data to estimate the 10-year cumulative cost of the two scenarios. The difference between these two indicate the potential budget impact associated with the application of the new ELITA guidelines in Europe, over the next 10 years, considering all HBV transplanted patients.

Additional analyses were performed to assess the following: 1. The impact of HBIG price to the budget impact results, applying the lowest and highest price reported within the 6 European countries used to estimate the HBIG price, 2. The variations in the number of incident LT patient, 3. Variations in the number of prevalent LT patient (±20% of base case), 4. The use of 1000 IU intramuscular (IM) or subcutaneous (SC) HBIG (327.6 Euro) every 2 weeks instead of 5000 IU IV HBIG (1,589.6 Euro) every 2 months, and 5. The use of 1000 IU IM or SC HBIG every 4 weeks instead of 5000 IU IV every 2 months. Further, to test the impact of a lower percentage of low-risk patients, an alternative scenario was tested assuming 70.0% low risk patients, 16.5% high-risk, and 13.5% special patients.

No human studies are presented in this manuscript; ethics approval or specific consent procedures were not required.

## Results

**FIGURE 1:**
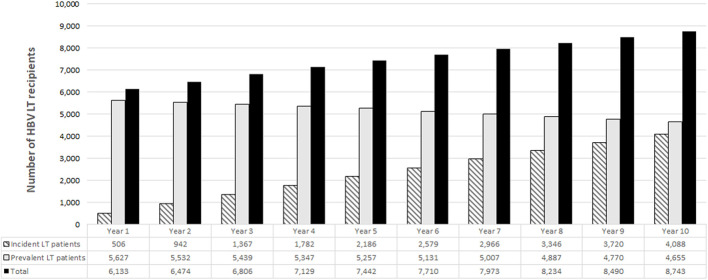
Target population estimated by the model.

The target population in the prediction model included both prevalent and incident cases, and consisted of 6,133 patients in the first year (Figure 1) that increased to 7,442 and 8,743 patients after 5 and 10 years from its implementation.

According to the historical protocol the cumulative costs were the following.

**FIGURE 2:**
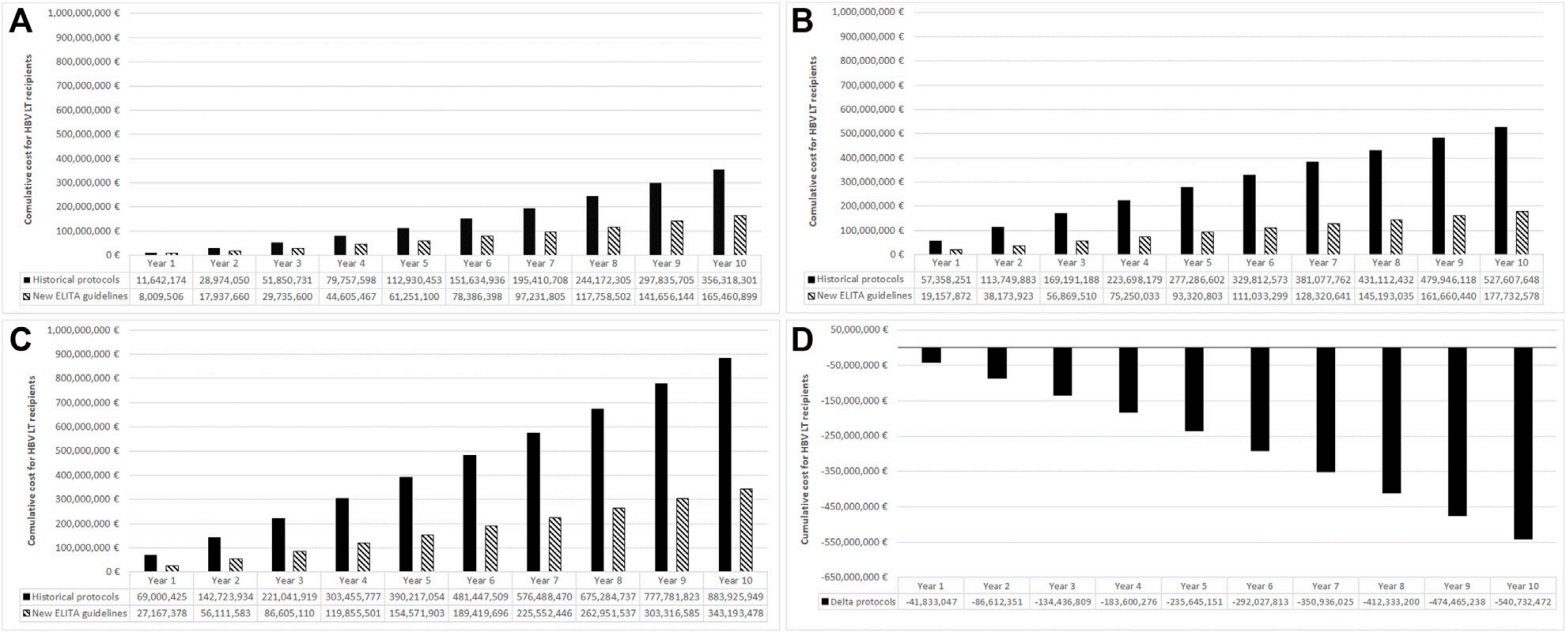
Total budget for the historical protocols and new ELITA treatment guidelines in incident (new) cases **(A)**, prevalent cases **(B)**, all cases **(C)**, and the associated budget impact **(D)**. Results from the European point of view and using 10 years time horizon.

- For incidental patients: 11.64 Million Euro after 1 year and 356.32 million Euro after 10 years (Figure 2A, black bars).
- For prevalent patients: 57.36 million Euro after 1 year and 527.61 million Euro after 10 years (Figure 2B, black bars).
- For prevalent + incident patients: 69.00 million Euro after the first year that increased to 883.93 million Euro after 10 years (Figure 2C, black bars)

The costs of the adoption of the ELITA guidelines, the dashed bars in Figure 2 panel A, B and C, was associated with a significant budget reduction and a cost saving of almost 41.83 million Euro at 1 year, 235.65 million Euro at 5 years, and 540.73 million Euro at 10 years (Figure 2D). The saving associated with new HBV post-LT prophylactic regimen was due to HIBG withdrawal after the first 4 weeks post LT in low and high risk populations, and to the increasing number of patients over time treated according to the ELITA guidelines.

The results of sensitivity analysis are reported in Table 2, and confirmed the significant cost saving associated to the adoption of the new ELITA guidelines. The use of 1000 IU IM or SC HBIG (327.6 Euro) every 4 weeks instead of 5000 IU IV HBIG (1,589.6 Euro) every 2 months was the parameter with the highest impact on the budget difference between the two scenarios, followed by HBIG price. The use of 1000 IU IM or SC HBIG every 4 weeks instead of 5000 IU IV HBIG every 2 months resulted in a cost saving of 252.31 million Euro at 10 years instead of 540.73 million Euro. Applying a price of 1,029.0 Euro per 5000 IU instead of 1,589.6 Euro, the model predicted a budget cut of 348.49 million Euro.

**TABLE 2:** Sensitivity analysis results.

Parameters	Value - base case	Budget impact—Base case	Value—sensitivity analysis	Budget impact—sensitivity analysis	Budget impact difference
New yearly (incident) cases of HBV LT patients, N	506	−540,732,472 €^a^	405	−502,636,429 €	−38,096,043 €
			607	−578,828,154 €	38,096,043 €
Number of prevalent HBV LT patients, N	5,627		4,501	−470,739,495	−69,992,976 €
			6,752	−610,711,633 €	69,979,161 €
HBIG IV, € per 5000 IU	1,589.6 €		1,029.0 €	−348,487,657 €	−192,244,815 €
Type and dose of HBIG	5000 IU IV every 2 months		1000 IU IM or SC HBIG every 2 weeks	−551,414,186 €	10,681,714 €
			1000 IU IM or SC HBIG every 4 weeks	−252,313,764 €	−288,418,708 €
Distribution of patients category included in ELITA protocol	82.0% (low risk)		70.0% (low risk)	−504,881,105 €	−35,851,367 €
	10.0% (high risk)		16.5% (high risk)		
	8.0% (special patients)		13.5% (special patients)		

^a^ Saving at 10 years in LT, récipients (incident + prevalent) using new ELITA, guidelines (overall cost € 343, 193, 478) instead of historical protocol (overall cost € 883, 925, 949).

## Discussion

New guidelines or international society recommendations provide up-to-date clinical evidence for improving clinical outcomes and managing patients. Unfortunately, economic impact analysis associated with the implementation of new guidelines or recommendations is rarely performed. Our study provides the budget impact analysis of the new strategies provided by ELITA for the management of liver-transplanted patients with Hepatitis B assuming that all prevalent cases (patients already transplanted) and incident cases (new patients undergoing LT) would be treated accordingly. Costs derived from the new ELITA guidelines were compared with those associated with historical protocols.

Based on our analysis, the implementation of the ELITA guidelines, resulted in a substantial cost saving which was possible thanks to an individualized short-term use of HBIG depending on the virological risk at the time of liver transplantation. In particular, according to the ELITA guidelines patients are considered at low or high risk if HBVDNA is undetectable (low risk) or positive at LT (high risk). Patients with low risk profile account for the vast majority of cases and are indicated to receive HBIG for the first 7 days after LT while high risk patients should be treated with HBIG for 1 year. Both low and high-risk patients are continued on NA alone after HBIG withdrawal.

Considering both prevalent and incident cases throughout Europe, the cost savings favored by the implementation of the ELITA clinical practice guidelines, was estimated at 235.65 million Euro at 5 years and 540.73 million Euro after 10 years. The cost saving is mainly associated to the reduction of HBIG use which produced a cost reduction of €9,538 per patient year both in low and high risk patients after the first year post LT. Assigning both incident and prevalent patients to new ELITA treatment guidelines provided the largest savings. The implementation of the ELITA guidelines to solely incident patients would also lead to significant cost savings, however the impact would become more significant after years due to the increasing number of patients treated with this new treatment protocol. Even the use of very low dose of HIBG, 1000 IU IM or SC HBIG every 4 weeks long term, was associated to a substantial saving (252.31 million Euro). In this scenario, the cost saving was €4,328 per patient year both in low and high risk patients after the first year post LT.

The results of this study are in accordance with the preliminary results reported in the original ELITA paper, however the current model which now includes prevalent cases, allows a more specific and accurate assessment of the potential overall economic impact. In fact, the estimated saving is higher to what assessed in previous analysis [3] as patients with HCC were not considered as a special population but received a prophylactic regimen based on their virological risk, similarly to decompensated cirrhotics.

An alternative prophylactic strategy, based on the use of NA without HBIG, has also been proposed by Fung and colleagues from Asia [2]. This treatment protocol would allow a greater reduction in treatment costs of 286.09 million Euro after 5 years and 647.73 million Euro after 10 years, compared to the historical treatment regimen. Furthermore, the approach proposed by Fung and colleagues compared favorably even with the new ELITA protocol, with around €50 million and €100 million savings at 5 and 10 years. Although such approach proved to be very effective and safe in Asia, new studies generated in western countries are needed before being accepted in European guidelines.

The study has some limitations. First, the analysis does not consider the clinical efficacy of the different scenarios (incidence of expected HBV recurrence). However, the available evidence suggests that the historical and new treatment protocols are associated with a similar efficacy that is an average 5% incidence of treatment failures [3]. Second, the analysis does not consider the adverse events associated with the historical protocols and ELITA guidelines. This approach is conservative considering the better safety profile associated to the lower use of HBIG in the new ELITA protocol. Third, the analysis assumed no treatment discontinuation in order to estimate the maximum economic impact associated with the new ELITA protocol. A different treatment adherence could in fact be observed when following historical protocols or new ELITA guidelines. In this case, a different clinical efficacy should be considered and a cost-effectiveness analysis should be conducted instead of a budget impact analysis. Fourth, the cost of HBIG IV infusion was not included in the analysis due to the high variability of cost and setting throughout Europe. However, this approach can be considered conservative due to the higher use of HBIG in the Historical scenario and the related higher cost associated to the infusions. Finally, the analysis assumed the use of Tenofovir Viread but not that of the more expensive Vemlidy. Since, the use of NA is the same in both historical protocols and ELITA guidelines; using a more or less expensive NA has no impact on the budget impact.

In conclusion, the new ELITA recommendations provide an individualized treatment prophylaxis of HBV patients based on virological risk profile at LT that would allow a significant cost reduction in Europe. The money saved would give the possibility to the healthcare system to invest in other technologies in order to improve the health of the population. New studies focusing on economic impact of ELITA guidelines in each European country could be of interest to provide specific information for local healthcare authorities and patients.

## Data Availability

The original contributions presented in the study are included in the article/Supplementary Material, further inquiries can be directed to the corresponding author.

## References

[1] Cholongitas E Papatheodoridis GV. High Genetic Barrier Nucleos(t)ide Analogue(s) for Prophylaxis from Hepatitis B Virus Recurrence after Liver Transplantation: a Systematic Review. Am J Transpl (2013) 13:353–62. 10.1111/j.1600-6143.2012.04315.x 23137006

[2] Fung J Wong T Chok K Chan A Cheung TT Dai JWC Long-term Outcomes of Entecavir Monotherapy for Chronic Hepatitis B after Liver Transplantation: Results up to 8 Years. Hepatology (2017) 66:1036–44. 10.1002/hep.29191 28370215

[3] Duvoux C Belli LS Fung J Angelico M Buti M Coilly A 2020 Position Statement and Recommendations of the European Liver and Intestine Transplantation Association (ELITA): Management of Hepatitis B Virus-Related Infection before and after Liver Transplantation. Aliment Pharmacol Ther (2020) 54(5):583–605. 10.1111/apt.16374 34287994

[4] Sullivan SD Mauskopf JA Augustovski F Jaime Caro J Lee KM Minchin M Budget Impact Analysis-Principles of Good Practice: Report of the ISPOR 2012 Budget Impact Analysis Good Practice II Task Force. Value Health (2014) 17(1):5–14. 10.1016/j.jval.2013.08.2291 24438712

[5] Adam R Karam V Cailliez V O Grady JG Mirza D Cherqui D 2018 Annual Report of the European Liver Transplant Registry (ELTR) - 50-year Evolution of Liver Transplantation. Transpl Int (2018) 31:1293–317. 10.1111/tri.13358 30259574

[6] Fraser SD Roderick PJ Casey M Taal MW Yuen HM Nutbeam D. Prevalence and Associations of Limited Health Literacy in Chronic Kidney Disease: a Systematic Review. Nephrol Dial Transpl (2013) 28:129–37. 10.1093/ndt/gfs371 23222414

[7] Ladin K Daniels A Osani M Bannuru RR. Is Social Support Associated with post-transplant Medication Adherence and Outcomes? A Systematic Review and Meta-Analysis. Transpl Rev (Orlando) (2018) 32:16–28. 10.1016/j.trre.2017.04.001 PMC5658266 28495070

